# Partners in crime? Radical scepticism and malevolent global conspiracy theories

**DOI:** 10.1007/s11229-024-04736-3

**Published:** 2024-08-30

**Authors:** Genia Schönbaumsfeld

**Affiliations:** Department of Philosophy, University of Southampton, Highfield Road, Southampton, SO17 1BF UK

**Keywords:** Radical scepticism, Global conspiracy theories, Local sceptical scenarios, Global sceptical scenarios, Particularism, Generalism, Descartes, Wittgenstein

## Abstract

Although academic work on conspiracy theory has taken off in the last two decades, both in other disciplines as well as in epistemology, the similarities between global sceptical scenarios and global conspiracy theories have not been the focus of attention. The main reason for this lacuna probably stems from the fact that most philosophers take radical scepticism very seriously, while, for the most part, regarding ‘conspiracy thinking’ as epistemically defective. Defenders of conspiracy theory, on the other hand, tend not to be that interested in undermining radical scepticism, since their primary goal is to save conspiracy theories from the charges of irrationality. In this paper, I argue that radical sceptical scenarios and global conspiracy theories exhibit importantly similar features, which raises a serious dilemma for the ‘orthodox’ view that holds that while we must respond to radical scepticism, global conspiracy theories can just be dismissed. For, if, as I will show, both scenarios can be seen to be epistemically on a par, then either radical sceptical scenarios are as irrational as global conspiracy theories or neither type of scenario is intrinsically irrational. I argue for the first option by introducing a distinction between ‘local’ and ‘global’ sceptical scenarios and showing how this distinction maps onto contemporary debates concerning how best to understand the notion of a ‘conspiracy theory’. I demonstrate that, just as in the case of scepticism, ‘local’ conspiracies are, at least in principle, detectable and, hence, epistemically unproblematic, while global conspiracy theories, like radical scepticism, are essentially invulnerable to any potential counterevidence. This renders them theoretically vacuous and idle, as everything and nothing is compatible with what these ‘theories’ assert. I also show that radical sceptical scenarios and global conspiracy theories face the self-undermining problem: As soon as global unreliability is posited, the ensuing radical doubt swallows its children – the coherence of the sceptic’s proposal or the conspiracy theorist’s preferred conspiracy. I conclude that radical sceptical scenarios and global conspiracy theories are indeed partners in crime and should, therefore, be regarded as equally dubious.

## Introduction

At the start of the pandemic, I received an email from a former undergraduate, who, upon hearing that I was ill with Covid, said, ‘Oh, so the virus *does* exist!’ That was not meant as a joke. When I asked him for an explanation, he offered up one of the global conspiracy theories currently in circulation: the virus and virus measures are all a smokescreen for The Global Microsoft Conspiracy – Bill Gates’ attempt to take over the world. I was surprised by this along a number of dimensions. First, this was a very bright student, showing that intelligence does not protect one from falling prey to believing outrageous falsehoods. Second, the student mentioned that he regarded global conspiracies as things to take seriously after having been exposed to Descartes’ Evil Demon argument in one of my epistemology classes. Third, when I tried to disabuse the student of this conspiracy, this proved a very difficult task for reasons similar to attempts to refute radical scepticism: whatever evidence is cited against the theory can be accommodated by proponents of the theory as being just what one would expect given the devious machinations of the conspirators (or the demon).

For example, I asked this student whether it really is plausible to think that virtually *all* media outlets and reputable scientific journals are in this conspiracy together, for it would take unprecedented levels of collusion, deception and coordination to make this possible. So much so, that this sounds exceptionally improbable. The student said that it’s not improbable, because all these channels are controlled and funded by Bill Gates. Such a response is actually not dissimilar to that of someone who is considering radical scepticism in epistemology and wondering whether his or her sources of information, including perception, are all controlled by Descartes’ Evil Demon or an Evil Scientist who has envatted his (or her) brain: highly improbable, but possibly true! Hence, what we seem to have here is an important similarity between radical scepticism and global conspiracy theories that could have significant implications for how we deal with both. For instance, are we entitled to write either off on a priori grounds because the theories are so improbable? Many people think so in the conspiracy case, but not that many philosophers do in the radical sceptical one. What should we make of this fact? Does it mean that we are rashly writing off conspiracy theories too?

Although academic work on conspiracy theory has taken off in the last two decades, both in other disciplines (e.g., in Politics, History, Psychology, Psychoanalysis, Sociology, Literary and Media Studies etc.; for an excellent overview see Butter & Knight, 2020) as well as in epistemology (Basham, 2003, 2006; Cassam, 2019a, b; Coady, 2006a, b, 2007; Clarke, 2006, 2007; Dentith, 2014, 2018a, b; Harris, 2018; Keeley, 1999, 2007; Pigden, 2006, 2007, 2017), the similarities between global sceptical scenarios and global conspiracy theories have not been the focus of attention^1^. The main reason for this lacuna probably stems from the fact that most philosophers take radical scepticism very seriously, while, for the most part, regarding ‘conspiracy thinking’ as epistemically defective (Baurmann & Cohnitz, 2021; Boudry, 2023; Cassam, 2019a, b; Goldberg, 2021; Harris, 2018; Levy, 2007; Napolitano 2021; Popper 2006; Spiegel, 2022). Defenders of conspiracy theory (Basham, 2003, 2006, 2018; Coady 2006a, b, 2007; Dentith 2018a, b; Hagen, 2018; Pigden, 2006, 2007, 2018), on the other hand, tend not to be that interested in undermining radical scepticism, since their primary goal is to save conspiracy theories from the charges of irrationality and paranoia.

If I am right, however, that radical sceptical scenarios and global conspiracy theories exhibit importantly similar features, then this could imply that both phenomena are epistemically on a par. Given that the majority of epistemologists believe that considering radical sceptical doubt constitutes a form of epistemic responsibility, while entertaining global conspiracy theories is thought irrational, these putative parallels raise a serious dilemma: either radical sceptical scenarios are as irrational as global conspiracy theories or neither type of scenario is intrinsically irrational. Both horns cause trouble for what one might call the ‘orthodox’ account that holds that while radical scepticism must be taken seriously, global conspiracy theories can be dismissed. My aim in this paper is to show that this dilemma gives us reason to reject the orthodox account and to be suspicious of *both* radical scepticism *and* global conspiracy theories.

My strategy is as follows. In the next sections, I introduce a distinction between ‘local’ and ‘global’ sceptical scenarios and show how this distinction maps onto contemporary debates concerning how best to understand the notion of a ‘conspiracy theory’. I will argue that, just as in the case of scepticism, ‘local’ conspiracies are, at least in principle, detectable and, hence, epistemically unproblematic, while global conspiracy theories, like radical scepticism, are essentially invulnerable to any potential counterevidence. I will show that this is precisely what makes them epistemically dubious and, in the end, as illusory as radical sceptical scenarios.

## The ‘Cliff Dive’

Long before the pandemic, in dark and mood-setting language, Basham (2001/2006,2) writes the following:

What if you are told that select Freemasons and a consortium of satellite groups are the secret masters of the planet? What if you are shown that if this is true the history of western civilization, down to minute details, becomes an *illuminated path*, an incredibly clear, cogent chronology? All it takes is a cognitive cliff dive into the dark, spooky waters of *conspiracy theory.* But few of us will go near that cliff. Even fewer will step to the edge and peer down at the abyss. Only the fewest step into the air and take the plunge. This is surprising. Why is contemporary conspiracy theory, with its seductive promise of hidden knowledge and genuine understanding, not the norm in human belief instead of the notorious exception? The temptation is obvious. Why the commonplace refusal, one so complete it’s as if many of us think *the cliff dive isn’t even a rational option?* As reason-obsessed philosophers we probably assume the answer lies in rational epistemology. We likely believe that it’s an easy task to show most conspiracy theories are *utterly unwarranted.* But is it? (Basham, 2006: 61)

Since Basham posed his rhetorical question, many more philosophers have joined the ranks of those who are willing to take the dive or at least to ‘peer down at the abyss’. This in itself is an interesting development. Before commenting on some of these newer trends, however, I want to juxtapose Basham’s evocation with the paradigmatic radical sceptical scenario in epistemology – Descartes’ conjuring of the Evil Demon:

I will therefore suppose that, not God, who is perfectly good and the source of truth, but some evil spirit, supremely powerful and cunning, has devoted all his efforts to deceiving me.^3^ I will think that the sky, the air, the earth, colours, shapes, sounds, and all external things are no different from the illusions of our dreams, and that they are traps he has laid for my credulity; I will consider myself as having no hands, no eyes, no flesh, no blood, and no senses, but yet as falsely believing that I have all these; I will obstinately cling to these thoughts, and in this way, if indeed it is not in my power to discover any truth, yet certainly to the best of my ability and determination I will take care not to give my assent to anything false, or to allow this deceiver, however powerful and cunning he may be, to impose upon me in any way^4^.

Both Descartes’ and Basham’s scenarios, then, posit ‘arch deceivers’ who have employed all their energies into ‘laying traps for one’s credulity’. And once such a scenario is actively entertained, nothing will be as it seems and all ‘external’ or ‘social’ reality turned into mere illusion.

Despite their clear structural similarities, however, these two scenarios are nevertheless treated very differently by their proponents. While Descartes is greatly perturbed by the doubts he has raised only as a thought experiment, Basham gives his scenario a positive spin, going as far as claiming that if we accept the mooted conspiracy theory, the history of western civilization becomes an ‘illuminated path’. What is more, while Descartes and subsequent generations of philosophers have emphasized the difficulty of laying his extreme doubt to rest, most philosophers, according to Basham, mistakenly believe that it’s a comparatively easy task to show that global conspiracy theories are ‘utterly unwarranted’.

In order to assess Basham’s claim that philosophers have merely fooled themselves into thinking that it is easy to show that global conspiracy theories are ‘utterly unwarranted’, we first need to ask how one would go about motivating such a global conspiracy theory in the first place. For, *prima facie*, it just looks like a fantasy plucked out of thin air (as, indeed, does the Evil Demon scenario). The arguments that Basham advances for the plausibility of the notion are, in fact, general sceptical ones. So, let’s take a closer look.

In ‘Living with the Conspiracy’, Basham makes two claims: (1) We have only limited grounds on which to claim positive warrant for our confidence in public institutions of information where critical interests of the dominant powers are at stake; and (2) abundant positive warrant exists to suspect public institutions of information are commonly used to deceive us in the pursuit of these interests (Basham, 2006: 67). It is precisely this positive warrant, Basham asserts, that ‘places many conspiracy theories in an entirely different league than the merely speculative schemes and concerns of global philosophical scepticism’ (ibid.).

Contrary to what Basham contends, however, I will show that these ‘leagues’ are not, in fact, very far apart. For, Basham’s line of reasoning closely parallels standard sceptical arguments in epistemology. In order to see this, one need only substitute the reliability of perception for ‘confidence in public institutions of information’. If so, we get: 1*) We have only limited warrant for believing that perception is generally reliable. 2*) Abundant positive warrant exists that perception often misleads us – the prevalence of perceptual illusions and hallucinations being cases in point. It is precisely on the grounds of (2*) that Descartes, for example, counsels, ‘Certainly, up to now whatever I have accepted as fully true I have learned either from or by means of the senses: but I have discovered that they sometimes deceive us, and prudence dictates that we should never fully trust those who have deceived us even once.’^5^ Basham draws exactly the same conclusion – since we have ample evidence of conspiratorial activity going on in society, we should be agnostic as to whether public institutions and sources of information are really reliable and not part of a ‘malevolent global conspiracy’.

The problem with this line of thought – as Descartes is to some extent aware (and which is why he brings in the dreaming and Demon arguments) – is that the possibility of perceptual error is not, by itself, enough to motivate a general distrust of the senses. And the same applies to Basham’s argumentative strategy: the fact that conspiracies sometimes occur is not a good reason to think that a malevolent global conspiracy currently has us in its grip. Indeed, the reverse is the case. That it’s possible for actual conspiracies to come to light and be known (i.e., conspiracies that are known facts, such as the Watergate scandal), implies that it cannot be the case that we are the victims of a truly global conspiracy. For, if we were, we would have no reason – no warrant – to believe that these actual conspiracies have occurred in the first place, as, in such a global conspiratorial scenario, we would not be able to take anything coming out of ordinary news sources or public institutions at face value. But since the fact that conspiracies, such as the Watergate scandal, have happened is supposed to motivate distrust in public institutions and sources of information and give us reason to think that ‘global conspiracy’ is a distinct possibility, this argument ends up undermining itself. Without prior trust in public institutions and news outlets, no conspiracy can be diagnosed as having definitively occurred, and without the certainty that some conspiracies have, in fact, occurred, there is no reason for a general distrust of public institutions and the reputable media.

Similarly, one is only able to diagnose a perceptual illusion as having occurred, if one does not start with a general distrust of sense-perception. For example, one can only determine that when one looks at a square tower from a distance, it will appear round, because one can trust one’s perception that from close-by it looks square, and there is a scientific explanation available that can tell one why it nevertheless appears round from some way off. If perception is generally assumed to be deceptive, as the sceptical argument contends, one could not make the judgment that perceptual appearances are sometimes misleading. All one could do would be to report, for instance, that at time t1 one has the impression that ‘thing 1’ that one sees is square, while at time t2 one has the impression that ‘thing 2’ that one sees is round. And since ‘thing 1’ and ‘thing 2’ might, for all one knows, be different things, one could not even conclude that one perceptual experience might be an accurate representation of the way things are, while the other might not. But if one cannot make this judgement, one is similarly unable to conclude that at time t2 one is being misled, for one’s perceptual experience at t2 would only be misleading if it were an experience of the same thing that one encountered at t1, so that one’s reports at t1 and t2 would turn out to be in conflict with each other. As long as one has no reason for assuming that one’s perceptual experiences of ‘thing 1’ and ‘thing 2’ are in fact experiences of the same physical object, however—which, of course, one would not if perception were generally defective (for then one would have no grounds for trusting one report more than the other; one would rather have to assume they are equally misleading)—there is no way of determining that a perceptual ‘illusion’ has in fact occurred^6^. All one could say is that one is having different perceptions at different times, but this, of course, does not suffice to allow one to infer that at time t2 one was misled, and, hence, that perceptual errors are possible^7^. In other words, perception must generally be taken to be reliable if an ‘argument from illusion’ is to be constructed. So, the attempt to use the possibility of perceptual illusion as a means of undermining the reliability of perception backfires, as one can only be certain that such an illusion or error has occurred, if one already starts by taking at least some perceptual experiences at face value^8^.

Exactly the same is true of using evidence of conspiratorial activity in society as a means of trying to motivate the thought that we could be victims of a global conspiracy. The ‘evidence of conspiratorial activity’ can only be taken as *evidence* if a malevolent global conspiracy is not thought systematically to be undermining all reputable sources of information, as it will otherwise also undermine the evidence of conspiratorial activity. Hence, the availability of evidence of conspiratorial activity does not entail, but rather *precludes* that we could be the victims of a malevolent global conspiracy.

## Aggregate arguments

The moral to draw from the previous discussion is that perceptual fallibility or localized institutional deception (such as happened in the Watergate scandal) is not by itself sufficient to motivate either ‘global’ sceptical scenarios – the thought that perception is never veridical – or malevolent global conspiracy theories (a conspiracy so global it would undermine virtually all our sources of information). Neither would a closely related move be very effective: that of arguing that things that have sometimes happened, could always happen. For example, to believe that because deception sometimes occurs (in various actual conspiracies or because I draw the wrong conclusions from my perceptual seemings), it is possible that deception could always occur and, hence, that none of my informational sources, including perception, may be trustworthy. I call this type of argument an ‘aggregate argument’, or the attempt to get to a ‘global’ sceptical, or conspiracy, scenario by way of ‘aggregating’ cases of ‘local’ error^9^. Basham is clearly making something like this move when he claims, on the basis of conspiratorial activity going on in society, that ‘there is a serious *prior probability* of global conspiracy’ (Basham, 2006: 95).

But this does not follow at all. That various ‘localized’ nefarious practices are going on does not give us reason to think that ‘the “world” as we know it today is an elaborate *hoax*’ (Basham, 2006: 94). It is a fallacy to think that what can sometimes happen, could always happen. From the fact that I can sometimes hit a bull’s eye in darts, it does not follow that I could always do this. From the fact that my child sometimes lies to me (for example, about being too ill to go to school), it does not follow that he could always be deceiving me. From the fact that people sometimes simulate pain, it does not make sense to suppose that they could always be simulating. Since if people didn’t generally complain, moan, grimace, express fear of the dentist etc. when they had toothache, for example, it would not be possible to choose *not* to express, or to *simulate*, these things either. Simulation or deception are parasitic on in principle veridicality. In short, from the fact that one can sometimes be wrong, it does not follow that one could always be wrong. Consequently, ‘local’ hoaxes or conspiracies cannot be aggregated into ‘global’ ones.

## Appeals to logical possibility

At this point in the discussion, retreats to logical possibility tend to be made. That is to say, given the failure of the previous two sceptical (or conspiratorial) strategies, defenders of scepticism or conspiracy theory may want to say something like this. It is at least logically possible that I have been captured in my sleep and turned into a brain-in-a-vat (BIV), so if I cannot rule this possibility out, how can I be sure that this is not how things are? Similarly, it is at least logically possible that at least one malevolent global conspiracy is in operation, so if I cannot rule this out, how do I know that this is not the case?

To be sure, one can imagine all sorts of things if one has nothing better to do. The thing is that nothing much follows from this. The mere fact that I can imagine a state of ‘envatment’ (or of being trapped in a malevolent global conspiracy) does not give me a real reason to suppose that I might actually be the victim of such a situation. Why not? Because, in the absence of a general argument designed to undermine the possibility of knowledge per se, I have no real grounds for thinking that the imagined scenario might be the actual one. For example, the state of current science is not yet advanced enough to make such envatment scenarios even empirically possible; there is no evidence of alien or robot activity etc. In other words, the thought that, despite all the evidence to the contrary (or the absence of evidence, as in the case of the global conspiracy theory), I might nevertheless be a BIV, purely because this is logically conceivable, is not yet to offer a reason in favour of this scenario: logical conceivability alone is not a form of evidence.

For, if something is not logically possible, then it need not even be investigated, but this does not imply that logical conceivability counts, by itself, as a reason that speaks in favour of a given scenario. Klein provides a good example to illustrate this point. In order to have sufficient evidential support for the claim that a particular person that we see is Publius, for instance, we don’t first need to be able to rule out an incredulous bystander’s idea that it might not be Publius, but rather Magicus’s dog, whom Magicus has transformed into an exact duplicate of Publius, unless we already have independent grounds for thinking that Magicus has that ‘ability, an opportunity and a motive’ (Klein, 2004: 174).^10^^,^^11^.

Similarly, if someone claims that the owls hooting outside my window have been stuffed by Bill Gates, then I don’t need to take this proposal seriously. Since unless someone presents actual evidence in favour of such a scenario, the mere mooting of this ‘logical possibility’ is not enough to motivate it. In the absence of any independent evidence for the ‘hypothesis’, the mere ‘raising’ of such a sceptical error-possibility is not enough to require one to investigate, or respond, to it^12^. Consequently, malevolent global conspiracy theories and BIV hypotheses remain scenarios that one need not take seriously, unless more is available than logical possibility alone.

## Epistemic steadfastness versus dogmatism

Some conspiracy, or sceptical, scenarios can also be ruled out on the basis of what we already know. For example, we don’t need to take the ‘lizard’ conspiracy theory seriously, because we already know that there are no such things as humanoid lizards. Similarly, Coady argues, ‘we may not know how we can know that the Second World War ended in 1945 when we are presented with a sufficiently vast and malevolent conspiracy theory, but we still do know this historical fact’ (Coady, 2006b: 169).

One might object that ruling out, or not considering, alternative scenarios to what one already knows looks like dogmatism, which constitutes an epistemic vice. But perhaps dogmatism is sometimes epistemically warranted, as Kripke (2011) has argued. The dogmatic view, according to Kripke, is that ‘since we know all sorts of things we should now make a resolution not to be swayed by any future evidence’ (Kripke, 2011: 45). In other words, on Kripke’s conception, if I know that P, then I know that any evidence against P is misleading and should resolve not to be influenced by it. Thus, I can protect my knowledge from sceptical onslaught by simply not considering the putative ‘evidence’ against my view. This, however, and as Kripke admits, seems paradoxical, since dogmatism – irrational attachment to a doctrine or proposition (Roberts & Wood, 2007) – is usually considered a bad thing; an epistemic vice. So, how can such a stance actually be knowledge-conducive rather than just a wilful and irrational ignoring of counterevidence?

Following Cassam (2019a), we might respond that in order for a person to have the right to be confident that P – rather than merely dogmatically confident that P – their confidence must be properly grounded. In other words, it must be based on the evidence for P and be guided by this evidence, rather than, say, the subject’s own prejudices: ‘Where P is just a dogma to which S is attached in such a way that they would still have been confident that P regardless of the evidence then S isn’t guided by the evidence and they don’t have the right to be confident. By the same token, S doesn’t know that P in these circumstances even if P, by some chance, turns out to be true’ (Cassam, 2019a: 109). That is to say, in the ‘good’ case – where I do actually know that P and my knowledge is supported in the right way by the evidence – P is not just a dogma (an irrationally held proposition), but something I do, in fact, know. Consequently, it is questionable, as Cassam himself suggests, whether relying on this knowledge should properly count as an instance of ‘dogmatism’ at all, rather than being, as Roberts and Wood (2007) call it, an epistemically virtuous species of ‘firmness’ (Cassam, 2019a: 113), something that they contrast with ‘flaccidity’ – being too easily swayed by any counter-proposal.

That seems right. So, what I would propose is to call relying on our knowledge in the good case ‘epistemic steadfastness’ rather than ‘dogmatism’. That is to say, if we are in the good case (where we do in fact know what we believe we know and have evidence for it), ignoring spurious ‘counterevidence’ is not a form of ‘dogmatism’; it is rather a type of epistemic steadfastness – of using the knowledge one already has to rule out counter-claims that one knows to have no basis given what one already knows.

Now, a sceptic or conspiracy theorist might push back against this notion by raising the question of how one can be confident that one is in the good case and not in an alternative sceptical, or conspiracy, scenario. That is to say, they may grant that in cases where we do, in fact, have knowledge, we are justified in being epistemically steadfast, but how can we know that we are in this privileged position and not merely believing that we are?

The answer to this question is contextual and will depend on the agent as well as on features of the agent’s environment. As we have already seen, if one’s belief is true and guided by the evidence, then, *ceteris paribus*, one has *prima facie* reason to be sceptical of any counterevidence. Nevertheless, even if I am an epistemically virtuous agent, whose cognitive and perceptual capacities are operating as normal and who avoids being swayed by dogma or bias, if I happen to find myself in a bad epistemic environment, where a lot of deceptive activity is going on, then I may, of course, no longer be able to rely on my ordinary knowledge. For example, all other things being equal, I would not normally have any reason to think that I am travelling through ‘barn-façade’ country, and, hence, would generally have no good grounds for distrusting my perceptual experiences of barns. If, however, it were known that along a certain stretch of countryside along the M4 motorway, there are barn-shaped objects that are not barns, put there as an artistic installation to remind travelling folk of their rural origins, then I ought not to trust that my perceptual experience of a barn will, in these circumstances, be veridical^13^. (But, of course, this does not imply that it is *never* veridical – no ‘aggregate arguments’.)

Similarly, if I live in Russia, where all the news programmes are controlled by the state, I should not remain epistemically steadfast in my view that Russia is only conducting a ‘special operation’ in Ukraine, if I come across evidence to the contrary by managing to tune in to the BBC World Service, for instance. In such a scenario, sticking to the ‘official story’ put out by the state, without bothering to examine contrary evidence, would not constitute epistemic steadfastness, but would rather be an instance of straightforward dogmatism^14^.

Conversely, if I happen to be travelling to Russia from the UK, I would have good epistemic reasons to be wary of the Russian state-controlled media and not to take their propaganda seriously. But this, *pace* Battaly (2018), would not constitute a form of epistemically legitimate ‘closed-mindedness’. Disregarding sources one knows to be spouting pernicious falsehoods is not closed-mindedness at all. Rather, the opposite is the case. In such a scenario, not listening to the propaganda constitutes a case of virtuous epistemic steadfastness: disregarding ‘evidence’ that one knows has been tinkered with.

## Interim conclusions

Let us take stock. So far we have seen that it is not easy to motivate global sceptical or conspiracy scenarios merely by focussing on various ‘local’ ones – by noting that sometimes our perceptual experiences cannot be taken at face value or that some actual conspiracies have been exposed in the past. Neither is the mooting of mere logical possibilities sufficient to motivate such sceptical error-possibilities. What is more, unless provided with very strong evidence to the contrary, rather than logical possibility alone, some sceptical and conspiracy scenarios can be ruled out on the basis of what one already knows.

Consequently, Basham is wrong to assert that ‘there is a serious *prior probability* of global conspiracy’ (Basham, 2006: 95). Various ‘local’ conspiracies may indeed be going on at various times, but whether they are is a purely empirical question for which one needs to present strong empirical evidence. What one cannot do is aggregate cases of local error or nefarious practice in order to infer that a malevolent global conspiracy, or sceptical, scenario, is (most probably) in operation.

In the next section, rather than focussing on the difficulty of motivating radical sceptical or global conspiracy scenarios, I will move on to a consideration of the internal faults that these scenarios can be discerned to possess. The aim is to show that the more a conspiracy scenario approximates to a radical sceptical one, the more problematic it becomes.

## Evidential invulnerability and falsification-resistance

Radical (or ‘global’) sceptical scenarios and their conspiratorial counterparts – malevolent global conspiracy theories – are very odd beasts. For, contrary to various forms of ‘local’ error – such as believing one is seeing real barns when travelling through ‘fake barn’ country or being the victim, say, of conspiratorial fraud – ‘global’ sceptical or conspiracy scenarios don’t just assume that we are wrong about various aspects of the epistemological landscape, they rather posit that there is a systematic mismatch between (almost) everything we believe about the world and the way this world actually is. And, if this were in fact so, then, even if it seemed otherwise, we would never have knowledge of anything; nor could we ever find out (even in principle) whether such a scenario obtained, as any form of evidence one could appeal to would itself be part of the ‘grand illusion’.

In this respect, radical sceptical scenarios and malevolent global conspiracy theories are very much alike. Since both scenarios posit an almost all-encompassing mismatch between how things seem and how they actually are, any appeal to empirical counterevidence would be futile. For example, if I start with the assumption that I’m in Descartes’ Evil Demon Scenario, then the fact that I appear to be perceiving ‘external’ objects is of no help. Since were I to say to the Demon whose deceptive machinations I am trying to confront, that he cannot currently be messing with my mind, as I am certain that I am sitting in my dressing gown by the fire, he would just laugh. For, how can I rule out that this all-powerful Demon is not just making things appear to me thus-and-so, when in fact, they are quite different, and I don’t even have eyes, ears and other bodily features? Similarly, how can I be certain that the absence of empirical evidence for a global conspiracy implies that there is not, in fact, a global conspiracy in operation, given that the incredibly powerful conspirators would go out of their way to make things seem that way to me?^15^

At this point in the discussion, it is useful to remind ourselves that we have already seen that these global error-possibilities are entirely unmotivated – they are nothing more than thought experiments (bare logical possibilities). What is more, it is only if we already *start* with the assumption that a malevolent global conspiracy (or sceptical scenario) is currently in operation that we are justified in asserting that an absence of evidence is not evidence of absence. And this raises a serious problem for both radical sceptical scenarios and global conspiracy theories.

For, normally, when proposing a theory – for example, in science – one wants the theory to be well-supported by the available evidence. In order for a theory to be capable of receiving evidential support, however, it cannot be the case that *whatever* state of affairs one assumes to obtain, it is compatible with what the theory asserts. For, if every state of affairs is compatible with what the theory asserts, then one might just as well say that no state of affairs is compatible with what the theory asserts and, hence, one would have neither evidential accord nor conflict here: everything and nothing is compatible with what the theory asserts. But, if so, this would render the ‘theory’ entirely vacuous and idle.

Both radical sceptical and global conspiracy scenarios fall foul of this problem. As we have just seen, if I assume that I am in a radical sceptical scenario, then whatever I believe is the case in the world will be compatible with the assumption that I am constantly being deceived by an Evil Demon (or having my brain manipulated by an Evil Scientist), and that nothing is as it appears. So, whatever counterevidence I might seek to invoke, it will not be able to falsify the ‘theory’ that I am constantly being deceived. Similarly, if I assume that a malevolent global conspiracy is currently in operation, then whatever evidence against such an all-encompassing plot as I might muster, it will not be able to falsify the contention that this ‘evidence’ has merely been fabricated by the conspirators in order to mislead me.

Now, while not everyone would agree with Popper that falsification-resistance implies that a proposed ‘theory’ is not a scientific one, the vacuity and idleness charges seem hard to avoid. For, if everything and nothing is compatible with my alleged ‘theory’, what is it that this theory is really saying or explaining? For example, what is the theory ruling in and what out? If I cannot draw such a distinction, because any state of affairs is compatible with what the theory ‘says’, then it appears difficult to see what sort of explanation of the world this theory could actually be offering.

It is for similar reasons that Wittgenstein (1969) believes that a ‘global’ doubt makes no sense, since genuine doubt presupposes certainty^16^. One can only properly call something into question, if there is something else that is not doubtful in respect of which whatever is doubtful is doubtful. If everything is doubtful, then nothing is and one is again back to not saying anything; to not being able to make any kind of genuine claim or theoretical assertion. So, it looks like evidential invulnerability comes at the cost of complete emptiness. A ‘theory’ that can accommodate any and every putative counterevidence seems not to be a theory, but an ultimately meaningless string of words.

## ‘Particularism’ and ‘Generalism’

What can we learn from this? First, that the closer a posited conspiracy theory comes to being a malevolent global one, the greater the danger it will end up falling foul of the vacuity charge. And this has some important implications for the currently raging debate between so-called ‘particularism’ and *soi disant* ‘generalism’ in the philosophy of conspiracy theory. ‘Particularism’ is the view that conspiracy theories must be examined or dismissed on their individual merits (or lack thereof), whereas ‘generalism’ is the notion that conspiracy theories exhibit some intrinsically bad features on the basis of which they can be dismissed as a class. This distinction roughly tracks non-evaluative and evaluative uses of the term ‘conspiracy theory’. Particularists regard the term ‘conspiracy theory’ as semantically neutral and as referring to any activity that happens to mention a conspiracy or people doing something in secret – even surprise birthday parties can fall into this category (Dentith, 2018b: 39) – while generalists use the term evaluatively to refer to an epistemically dubious theory involving a conspiracy.

Interestingly, and as Boudry and Napolitano (2023) have recently pointed out, while particularists vocally nail their colours to the particularist mast (Basham, 2018; Dentith, 2018b; Coady, 2018; Pigden, 2018), self-proclaimed generalists are actually rather hard to find^17^. And this is, of course, because, contrary to what some particularists claim, even those who favour evaluative uses of ‘conspiracy theory’, do not deny that some conspiracies have actually happened (Watergate etc.). So, if ‘conspiracy theory’ just means, as particularists contend, ‘any explanation of a historical event that involves a conspiracy’, then, by definition, it’s impossible *not* to be a particularist (Boudry & Napolitano, 2023: 23), as, naturally, no one would deny that conspiracies have historically occurred. But this would then be a purely nominal victory for particularism^18^. For these reasons, Boudry and Napolitano suggest we retire the terms ‘particularism’ and ‘generalism’ altogether, as they no longer seem to serve a useful purpose and instead sow division where there is, perhaps, more agreement than this distinction would suggest.

On the basis of what I have argued so far, I propose something different (though I largely agree with Boudry and Napolitano). We can be particularists about ‘local’ conspiracy theories that are empirically well-motivated and plausible (rather than just logically possible) and sceptical generalists about more ‘global’ conspiracy theories that assume near-omnipotent agents able to dupe us about almost anything^19^. What is more, the more a ‘local’ conspiracy theory moves towards becoming akin to a radical sceptical one – for example, because particular conspiracy theorists can only accommodate the counterevidence to their view by positing an ever greater conspiracy and ever greater deception – the less plausible it becomes. For, such global conspiracy theories not only face the vacuity charge discussed in the previous section, they are also confronted by a related, self-undermining problem, which I discuss next^20^.

## The self-undermining problem

If global conspiracy theorists are right that most channels of information that we normally rely on (e.g., all of the mainstream media, all reputable academic journals, all academic research institutions etc.) are in fact, dubious, how can they insulate their own version of events from this far-reaching doubt? In other words, how can believers in the Global Microsoft Conspiracy be confident, for example, that they haven’t fallen prey to a different set of conspirators – Freemasons, say – who are so powerful as to be able to make it seem that the Global Microsoft Conspiracy is true? That is to say, how can a global conspiracy theorist rule out that their favoured conspiracy isn’t a sham too?

It seems that as soon as we take it to be plausible that *most* of the information that we receive cannot be trusted, we face a problem of radical *underdetermination*: We can no longer be sure that our preferred conspiracy theory is true, for if nothing is as it seems, any number of competing conspiracy theories that we haven’t even considered could be currently in operation! So, again, it appears that entertaining a global conspiracy theory is very similar to considering a radical sceptical scenario – as soon as we do this, we can no longer privilege our own perspective or save it from epistemic devastation.

For example, say I ‘doubt’ in ordinary circumstances where nothing unusual has occurred that I have hands. How could I respond to such a radical doubt? If, in such an ordinary context (i.e., not a context where my hands might have been amputated, for instance), I could nevertheless be ‘wrong’ about having hands, this would be no ordinary ‘mistake’, but would rather constitute what Wittgenstein, in *On Certainty* (OC), calls ‘an annihilation of all yardsticks’ (OC § 492). For, while a mistake does not call into question the intelligibility of the entire practice in which one is participating – but only one’s own competence – the notion that I could be ‘wrong’ about the most fundamental things undermines the coherence of the very practices on which my expression of doubt at the same time depends (since if there were no such practice, there would be no such doubt): ‘If you are not certain of any fact, you cannot be certain of the meaning of your words either’ (OC § 114).

That your words mean what they do is also a fact about them. So, if everything is uncertain, as the sceptic supposes, then ‘meaning’ (including the meaning of the sceptic’s own words) would be undermined along with everything else: ‘If my name is *not* L.W., how can I rely on what is meant by “true” and “false”?’ (OC § 515). That is to say, if I cannot be certain of what my own name is, or whether the computer in front of me is not a simulation, neither can I be certain of what ‘true’ or ‘false’ mean. Hence, it’s not just that scepticism is *unstateable* – but might, for all that, be true (as some philosophers believe^21^) – it is rather that, if Wittgenstein is right, it is no longer clear what the sceptic’s ‘thought’ can even amount to: ‘So is the *hypothesis* possible that all the things around us don’t exist? Would that not be like the hypothesis of our having miscalculated in all our calculations?’ (OC § 55) For, a ‘miscalculation’ in *all* our calculations is not, as it were, just an ‘aggregated’ mistake, but rather implies that we have never *calculated* at all – that nothing that we have ever done counts as an instance of *calculating*. Similarly, if the hypothesis is possible that there are no physical objects, then we are supposing that we have *never* encountered such a thing. But, if so, the sceptic owes us an explanation of what ‘calculation’ or ‘physical object’ really means, and of what it is, that we allegedly cannot do (or know). In the absence of such an explanation, we need not accept the sceptic’s challenge that unless we can show in advance that we are not radically mistaken about everything, we aren’t entitled to our ordinary knowledge claims.

Similarly, Basham (2006) is wrong to think that we first need to justify ‘basic claims’ about how conspiratorial society is before we are entitled (in a functioning democracy), to trust the mainstream media or government institutions. To be sure, some of these institutions may sometimes lead us astray, so one should not uncritically accept everything coming out of these sources, but it does not follow from this that it makes sense to suppose that they could always be leading us astray (no aggregate arguments). What is more, and as we have just seen, if we assume that they could always (or predominantly) be leading us astray – for example, because we are supposing that *all* historical records have been forged – then we ought to abandon our confidence in our preferred conspiracy theory too, as nothing will then be immune to this global doubt. So, the global conspiracy theorist, like the radical sceptic, cannot have her cake and eat it too: for either we are confronted by only a localized conspiracy, in which case we will be able to use ordinary sources of information to see through it, or else we are assuming a malevolent, global conspiracy, in which case, whatever the details of this conspiracy theory – it will be undermined too. To speak with Wittgenstein, if you are not certain of any (or most) facts, then you cannot be certain of your conspiracy theory either!

## Conclusion

Let us now summarize our results. I have argued for the following claims:

i) If we start by assuming global perceptual or informational unreliability, we will be unable to construct arguments from perceptual illusion (or from conspiratorial activity). Since such arguments are drafted in to *motivate* global sceptical or conspiracy scenarios, however, their grounds are undercut unless we grant that we are *not* in a global sceptical or conspiracy scenario.
ii) Aggregate arguments are fallacious. Consequently, one cannot argue that because error is sometimes possible, therefore error could always be possible.
iii) Logical possibility alone is not a form of evidence. Hence, unless independent evidence is available that speaks in favour of a particular sceptical or conspiratorial error-possibility, I need not take it seriously.
iv) If one is in the good case, one can rely on what one already knows, in order to rule out certain sceptical or conspiratorial hypotheses.
v) Radical sceptical scenarios and malevolent global conspiracy theories are evidentially invulnerable and falsification resistant. This renders them theoretically vacuous and idle, as everything and nothing is compatible with what these ‘theories’ assert.
vi) Radical sceptical scenarios and malevolent global conspiracy theories face the self-undermining problem: As soon as global unreliability is posited, the ensuing radical doubt swallows its children – the coherence of the sceptic’s proposal or the conspiracy theorist’s preferred conspiracy.

It follows from this that local sceptical or conspiracy scenarios are epistemically unproblematic in the sense that they are not invulnerable to evidence and are in principle detectable. Consequently, if we try hard enough, we can in principle find out whether such scenarios obtain or not. Global sceptical or conspiracy scenarios, on the other hand, are evidentially invulnerable, falsification-resistant and self-undermining. For these reasons, we need not take them seriously.

If this is right, then it implies that we should reject the orthodox view mentioned in the introduction. The structural similarities between radical sceptical scenarios and malevolent global conspiracy theories show that these scenarios are indeed partners in crime. So, if we can be persuaded that global conspiracy scenarios are epistemically problematic and should, therefore, be rejected, we ought to be able to see that exactly the same is true of radical sceptical scenarios. And while this does not constitute a refutation of global doubt^22^, as such a thing is in principle impossible given said evidential invulnerability and falsification-resistance, it does show that making concessions to radical scepticism may be less necessary and less of a badge of honour than most contemporary epistemologists suppose.^23^

## Footnotes

1 There is some interesting discussion in Basham (2003); Coady (2006b); Keeley (2006), to which I shall return below.

2 I will be citing from the paper reprinted in Coady, 2006a, not from the 2001 version, first published in *The Philosophical Forum* (Vol. 32 No. 3, 2001), 265 − 80.

3 It does not matter for the purposes of this paper whether we take the deceiver to be a hypothetical demon or an imagined evil scientist who has envatted our brains.

4 Descartes (2008:16–17).

5 Descartes (2008: 13).

6 I am here drawing on Schönbaumsfeld, 2016, ch. 2.

7 What if we suppose that, at t1, I have the impression that thing 1 that I see is square, at t2 I have the impression that thing 2 that I see is round, and between t1 and t2 I have the impression that thing 1 hasn’t changed shape or been replaced – wouldn’t we then have to conclude that a perceptual illusion has in fact occurred? We might indeed conclude in such a case that a perceptual illusion has occurred (as we might also in the other scenarios), but the relevant point is that we can only draw this conclusion because we are taking our impression that thing 1 has not changed between t1 and t2 (or been replaced) to be veridical, which confirms what I was trying to show: namely, that we cannot conclude that a perceptual error has occurred unless we are willing to grant that some of our sense-perceptions can be veridical. If we don’t, then we certainly cannot grant that we can take our impression that thing 1 hasn’t changed between t1 and t2 at face value (and hence we could not conclude that a perceptual error has occurred).

8 This has nothing to do with the question of whether perceptual error is always detectable. It may not be detectable in any given case. The point is rather that if I start with the assumption that *all* perceptions are unreliable, then I can *never* formulate an argument from illusion, as such an argument presupposes that some of our perceptions are reliable (e.g., that the tower has not changed shape and now looks square). Hence, I cannot use an argument from illusion in order to undermine the very thing the argument itself presupposes: the reliability of some of our sense-perceptions.

9 See Schönbaumsfeld, 2016, ch. 2, for further discussion.

10 Compare Battaly (2018).

11 The relevant point here is that we need *independent* grounds, i.e., grounds that are independent of already having accepted the hypothesis that it might be Magicus’s dog. Basham (2018); Dentith (2018b) sometimes write as if having the ability, an opportunity and a motive are by themselves sufficient. But if there is no independent evidence that a crime has been committed, then it is utterly irrelevant whether someone fulfils the aforementioned criteria. For example, if a putative murder is established to have been suicide, then the fact that the deceased’s brother would have had the ability, an opportunity and a motive to kill the deceased is beside the point. What is more, whether the death was a suicide or not cannot be determined by appealing to the question of who might have had the ability, an opportunity and a motive to kill the deceased, but must rather be settled via a pathology exam. Similarly, in the absence of independent, empirical evidence for this thesis (and not just reliance on ‘errant data’), the hypothesis that 9/11 was an inside job cannot be motivated by claiming that the US government would have had the ability, an opportunity and a motive to do something like this.

12 For more on unmotivated sceptical error-possibilities, see Pritchard (2012).

13 For further discussion, see Schönbaumsfeld, 2016, ch. 2.

14 Even if citizens are, perhaps, less to blame for this given that this counterevidence is not easily available.

15 Or to put it in Rumsfeld’s infamous words, absence of evidence is not evidence of absence.

16 For more on this, see Schönbaumsfeld, 2016, ch. 4.

17 As Boudry and Napolitano also highlight, there are those whom particularists accuse of being generalists, even though these philosophers would not describe themselves in this way (see, e.g., Clarke, 2023). Stokes (2018) defends what he calls a ‘reluctant particularism’.

18 Also compare Räikkä 2023.

19 Although Basham (2018) now calls himself a particularist, in earlier work he was quite happy to grant that some conspiracy theories can be dismissed on purely ‘internal’ grounds: ‘We ought to be sceptical of conspiracy theories when they suffer various *internal faults.* These include problems with self-consistency, explanatory gaps, appeals to unlikely or obviously weak motives and other unrealistic psychological states, poor technological claims and the theory’s own incongruencies with observed facts it grants (including failed predictions)’ (Basham, 2006: 72).

20 This self-undermining problem is not identical to the one I discuss in section II, but they are related.

21 A view that Stroud seemed to defend (in conversation).

22 For a full anti-sceptical strategy, see Schönbaumsfeld, 2016.

23 The author would like to thank two anonymous reviewers for their comments on an earlier draft of this paper.

## References

[CR1] Basham, L. (2003). Malevolent global conspiracies. *Journal of Social Philosophy*, *34/1*, 91–103.

[CR2] Basham, L. (2006). Living with the conspiracy. In D. Coady (Ed.), *Conspiracy Theories* (pp. 61–76).

[CR3] Basham, L. (2018). Conspiracy theory particularism, both moral and epistemic, versus generalism. In M R. X. Dentith (Ed.), *Taking Conspiracy Theories Seriously* (pp. 39–58).

[CR4] Battaly, H. (2018). Can closed-mindedness be an intellectual Virtue? *Royal Institute of Philosophy Supplement*, *84*, 23–45.

[CR5] Baurmann, M., & Cohnitz, D. (2021). Trust no one? The (social) epistemological consequences of belief in conspiracy theories. In S. Bernecker, A. K. Flowerree & T. Grundmann (Eds.), *The Epistemology of Fake News* (pp. 334–357). Oxford University Press.

[CR6] Boudry, M., & Napolitano, G. (2023). Why we should stop talking about Generalism and Particularism: Moving the debate on Conspiracy theories Forward. *Social Epistemology Review and Reply Collective*, *12*(9), 22–26.

[CR7] Butter, M., & Knight, P. (Eds.). (2020). *The Routledge Handbook of Conspiracy theories*. Routledge.

[CR8] Cassam, Q. (2019a). *Vices of the mind*. Oxford University Press.

[CR9] Cassam, Q. (2019b). *Conspiracy theories*. Polity.

[CR10] Clarke, S. (2006). Conspiracy Theories and Conspiracy Theorizing. In Coady (Ed.), *Conspiracy Theories* (pp. 77–92).

[CR11] Clarke, S. (2007). Conspiracy theories and the internet: Controlled demolition and arrested development. *Episteme*, *4*(2), 167–180.

[CR12] Clarke, S. (2023). Two problems with the generalist-particularist distinction in the philosophy of Conspiracy Theory and why I’m not a generalist. *Social Epistemology Review and Reply Collective*, *12*(8), 54–60.

[CR13] Coady, D. (Ed.). (2006a). *Conspiracy theories– the philosophical debate*. Ashgate.

[CR14] Coady, D. (2006b). Conspiracy Theory and Official Stories. In D. Coady (Ed.), *Conspiracy Theories* (pp. 115–128).

[CR15] Coady, D. (2007). Are conspiracy theorists Irrational? *Episteme*, *4*(2), 193–204.

[CR16] Coady, D. (2018). Anti-Rumour Campaigns and Conspiracy Baiting as Propaganda. In M R. X. Dentith (Ed.), *Taking Conspiracy Theories Seriously* (pp. 316–344).

[CR18] Dentith, M. (2014). *The philosophy of conspiracy theories*. Palgrave.

[CR19] Dentith, M. (Ed.). (2018a). *Taking conspiracy theories seriously*. Rowman and Littlefield.

[CR20] Dentith, M. R. X. (2018b). When Inferring to a Conspiracy Might be the Best Explanation. In M R. X. Dentith (Ed.), *Taking Conspiracy Theories Seriously* (pp. 28–65).

[CR17] Descartes, R. (2008). *Meditations on First Philosophy (with selections from the objections and replies), trans. Michael Moriarty*. Oxford University Press.

[CR21] Goldberg, S. (2021). Fake news and epistemic rot; or, why we are all in this together in Sven Bernecker. In A. K. Flowerree, & T. Grundmann (Eds.), *The epistemology of fake news*. Oxford University Press.

[CR22] Hagen, K. (2018). Conspiracy theories and the paranoid style: Do conspiracy theories posit implausibly vast and evil conspiracies? *Social Epistemology*, *32*(1), 24–40.

[CR23] Harris, K. (2018). What’s epistemically wrong with conspiracy theorising? *Royal Institute of Philosophy Supplement*, *84*, 235–257.

[CR24] Keeley, B. (1999). Of conspiracy theories. *Journal of Philosophy*, *96*, 109–126.

[CR25] Keeley, B. (2006). *Nobody* Expects the Spanish Inquisition! More Thoughts on Conspiracy Theory. In D. Coady (Ed.), *Conspiracy Theories* (pp. 107–114).

[CR26] Keeley, B. (2007). God as the Ultimate Conspiracy Theory. *Episteme*, *4*(2), 135–149.

[CR27] Klein, P. (2004). Closure matters: Academic scepticism and Easy Knowledge. *Philosophical Issues (Epistemology)*, *14*, 165–184.

[CR28] Kripke, S. (2011). On two paradoxes of Knowledge. In *Philosophical troubles volume 1* (pp. 27–51). Oxford University Press.

[CR29] Levy, N. (2007). Radically socialized knowledge and conspiracy theories. *Episteme*, *4*(2), 181–192.

[CR30] Napolitano, M. G. (2021). Conspiracy Theories and Evidential Self-Insulation. In *The Epistemology of Fake News* (pp. 82–105).

[CR31] Pigden, C. R. (2006). Popper Revisited, or What is Wrong with Conspiracy Theories? In Coady (Ed.), *Conspiracy Theories* (pp. 13–17).

[CR32] Pigden, C. R. (2007). Conspiracy Theories and the Conventional Wisdom’ in *Episteme*, *4*(2), 219–232.

[CR33] Pigden, C. R. (2017). Are conspiracy theorists epistemically vicious? In K. Lippert-Rasmussen, K. Brownlee, & D. Coady (Eds.), *A companion to Applied Philosophy*. Wiley Blackwell.

[CR34] Pigden, C. R. (2018). Conspiracy Theories, Deplorables, and Defectibility: A Reply to Patrick Stokes. In M R. X. Dentith (Ed.), *Taking Conspiracy Theories Seriously* (pp. 371–395).

[CR35] Popper, K. (2006). The Conspiracy Theory of Society. In D. Coady (Ed.), *Conspiracy theories. The philosophical debate* (pp. 13–17). Ashgate.

[CR36] Pritchard, D. (2012). *Epistemological disjunctivism*. Oxford University Press.

[CR37] Räikkä, J. (2023). *Why a pejorative definition of ‘Conspiracy Theory’* need not be unfair. *Social Epistemology Review and Reply Collective*, *12*(5), 63–71.

[CR39] Roberts, R. C., & Wood, W. J. (2007). *Intellectual virtues: An essay in Regulative Epistemology*. Oxford University Press.

[CR40] Schönbaumsfeld, G. (2016). *The illusion of doubt*. Oxford University Press.

[CR42] Spiegel, T. J. (2022). Verschwörungstheorien und das Erbe der Aufklärung: Auf den Schultern von Scheinriesen. *Deutsche Zeitschrift für Philosophie*, *70*(2), 253–273.

[CR41] Stokes, P. (2018). Conspiracy Theories and the Perils of Pure Particularism. In M R. X. Dentith (Ed.), *Taking Conspiracy Theories Seriously* (pp. 66–89).

[CR43] Wittgenstein, L. (1969). *On Certainty* (Eds. G. E. M. Anscombe and G. H. von Wright, Trans. Anscombe and D. Paul). Blackwell.

